# Di-*tert*-butyl 2,2′-[9*H*-fluorene-9,9-diylbis(*p*-phenyl­ene­oxy)]diacetate

**DOI:** 10.1107/S1600536810022579

**Published:** 2010-06-18

**Authors:** Kiramat Shah, Sammer Yousuf, Muhammad Raza Shah, Seik Weng Ng

**Affiliations:** a H.E.J. Research Institute of Chemistry, International Center for Chemical and Biological Sciences, University of Karachi, Karachi 75270, Pakistan; b Department of Chemistry, University of Malaya, 50603 Kuala Lumpur, Malaysia

## Abstract

In the title mol­ecule, C_37_H_38_O_6_, the non-fused C atom belonging to the five-membered ring of the fluorene system is connected to two *p*-phenyl­ene rings, the rings opening up the C_ar­yl_–C—C_ar­yl_ angle to 113.1 (1)°. The four-atom –O–CH_2_–C(=O)–O– chain between the *p*-phenyl­ene ring and the *tert*-butyl group assumes a more regular W-shaped conformation for one substituent [O—C—C—C torsion angle = 171.9 (2)°] but a less regular W-shaped conformation for the other [torsion angle = 147.4 (2)°].

## Related literature

For the application of the title compound as a dissolution inhibitor for protecting photosensitive poly-benzoxazoles, see: Ogura *et al.* (2009 ▶).
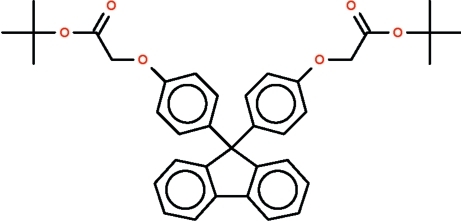


## Experimental

### 

#### Crystal data

C_37_H_38_O_6_
*M* *_r_* = 578.67
Monoclinic, 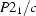

*a* = 15.6527 (8) Å
*b* = 11.9466 (6) Å
*c* = 17.8218 (9) Å
β = 107.109 (1)°
*V* = 3185.1 (3) Å^3^
*Z* = 4
Mo *K*α radiation
μ = 0.08 mm^−1^
*T* = 293 K
0.45 × 0.25 × 0.15 mm

#### Data collection

Bruker SMART APEX CCD diffractometer
21641 measured reflections
7322 independent reflections
4525 reflections with *I* > 2σ(*I*)
*R*_int_ = 0.033

#### Refinement

*R*[*F* ^2^ > 2σ(*F* ^2^)] = 0.051
*wR*(*F* ^2^) = 0.161
*S* = 1.00
7322 reflections
388 parameters
H-atom parameters constrained
Δρ_max_ = 0.20 e Å^−3^
Δρ_min_ = −0.18 e Å^−3^

### 

Data collection: *SMART* (Bruker, 2002 ▶); cell refinement: *SAINT* (Bruker, 2002 ▶); data reduction: *SAINT*; program(s) used to solve structure: *SHELXS97* (Sheldrick, 2008 ▶); program(s) used to refine structure: *SHELXL97* (Sheldrick, 2008 ▶); molecular graphics: *X-SEED* (Barbour, 2001 ▶); software used to prepare material for publication: *publCIF* (Westrip, 2010 ▶).

## Supplementary Material

Crystal structure: contains datablocks global, I. DOI: 10.1107/S1600536810022579/zs2047sup1.cif

Structure factors: contains datablocks I. DOI: 10.1107/S1600536810022579/zs2047Isup2.hkl

Additional supplementary materials:  crystallographic information; 3D view; checkCIF report

## Figures and Tables

**Fig. 1.**
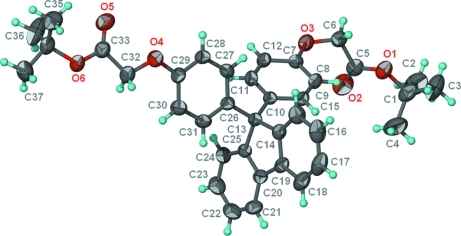
Thermal ellipsoid plot (Barbour, 2001) of C37H38O6 at the 50% probability level. Hydrogen atoms are drawn as spheres of arbitrary radius.

## supplementary crystallographic information

### Comment

9,9-Bis[4-(*tert*-butoxycarbonylmethyloxy)phenyl]fluorene (Scheme I) is described in the context of its function as a 'dissolution inhibitor' for protecting the photosensitive poly(benzoxazole)s used for protecting chips (Ogura *et al.*, 2009). The carbon atom belonging to the five-membered fluorenyl ring is connected to two *p*-phenylene rings which open up the C_aryl_–*C*–C_aryl_ angle to 113.1 (1) ° but the rings have to be rotated by 57.9 (1)°. Of the two four-atom –O–CH_2_–C(═ O)–O– chains between the *p*-phenylene ring and the *tert*-butyl group, one assumes a more regular *W*-shaped conformation [O–C–C–C torsion angle 171.9 (2)°] whereas the other assumes a less regular *W*-shaped conformation for the other [torsion angle 147.4 (2)°] (Fig. 1).

### Experimental

9,9-Bis(4-hydroxyphenyl)fluorene (0.5 g, 1.4 mmol) was dissolved in acetone (25 ml) to give a clear solution. Potassium carbonate (0.7 g, 5 mmol) was added and the mixture stirred for an hour. *tert*-Butylbromo acetate (1 ml, 5.6 mmol) was added and stirring continued overnight. The mixture was filtered, prismatic crystals separating from the solution in 80% yield.

### Refinement

Carbon-bound H-atoms were placed in calculated positions [C–H = 0.93–0.97 Å, *U*(H) = 1.2–1.5*U*_eq_(C)] and were included in the refinement in the riding model approximation.

### Crystal data

**Table:**

C_37_H_38_O_6_	*F*(000) = 1232
*M**_r_* = 578.67	*D*_x_ = 1.207 Mg m^−^^3^
Monoclinic, *P*2_1_/*c*	Mo *K*α radiation, λ = 0.71073 Å
Hall symbol: -P 2ybc	Cell parameters from 3717 reflections
*a* = 15.6527 (8) Å	θ = 2.2–21.9°
*b* = 11.9466 (6) Å	µ = 0.08 mm^−^^1^
*c* = 17.8218 (9) Å	*T* = 293 K
β = 107.109 (1)°	Block, colorless
*V* = 3185.1 (3) Å^3^	0.45 × 0.25 × 0.15 mm
*Z* = 4	

### Data collection

**Table:**

Bruker SMART APEX CCD diffractometer	4525 reflections with *I* > 2σ(*I*)
Radiation source: fine-focus sealed tube	*R*_int_ = 0.033
graphite	θ_max_ = 27.5°, θ_min_ = 2.1°
ω scans	*h* = −12→20
21641 measured reflections	*k* = −15→15
7322 independent reflections	*l* = −23→23

### Refinement

**Table:**

Refinement on *F*^2^	Primary atom site location: structure-invariant direct methods
Least-squares matrix: full	Secondary atom site location: difference Fourier map
*R*[*F*^2^ > 2σ(*F*^2^)] = 0.051	Hydrogen site location: inferred from neighbouring sites
*wR*(*F*^2^) = 0.161	H-atom parameters constrained
*S* = 1.00	*w* = 1/[σ^2^(*F*_o_^2^) + (0.0795*P*)^2^ + 0.3146*P*] where *P* = (*F*_o_^2^ + 2*F*_c_^2^)/3
7322 reflections	(Δ/σ)_max_ = 0.001
388 parameters	Δρ_max_ = 0.20 e Å^−^^3^
0 restraints	Δρ_min_ = −0.18 e Å^−^^3^

### Fractional atomic coordinates and isotropic or equivalent isotropic displacement parameters (Å^2^)

**Table:**

	*x*	*y*	*z*	*U*_iso_*/*U*_eq_	
O1	0.96192 (9)	0.50272 (11)	0.17980 (8)	0.0660 (4)	
O2	0.90616 (13)	0.40493 (15)	0.26336 (10)	0.0924 (5)	
O3	0.74271 (9)	0.40436 (10)	0.14068 (8)	0.0602 (4)	
O4	0.18694 (9)	0.79726 (14)	0.01378 (8)	0.0684 (4)	
O5	0.02575 (10)	0.72090 (14)	−0.06079 (10)	0.0831 (5)	
O6	−0.03695 (9)	0.82951 (14)	0.01154 (9)	0.0735 (4)	
C1	1.05054 (14)	0.53058 (19)	0.23489 (14)	0.0734 (6)	
C2	1.10416 (18)	0.4244 (2)	0.25468 (17)	0.0992 (9)	
H2A	1.1106	0.3917	0.2075	0.149*	
H2B	1.0740	0.3728	0.2794	0.149*	
H2C	1.1622	0.4411	0.2898	0.149*	
C3	1.08798 (19)	0.6088 (3)	0.18602 (19)	0.1086 (10)	
H3A	1.0953	0.5692	0.1414	0.163*	
H3B	1.1449	0.6367	0.2172	0.163*	
H3C	1.0476	0.6704	0.1684	0.163*	
C4	1.0403 (2)	0.5896 (3)	0.30693 (18)	0.1131 (10)	
H4A	1.0070	0.6575	0.2914	0.170*	
H4B	1.0983	0.6070	0.3418	0.170*	
H4C	1.0090	0.5417	0.3332	0.170*	
C5	0.90208 (14)	0.43879 (17)	0.19930 (13)	0.0614 (5)	
C6	0.82649 (13)	0.41483 (17)	0.12614 (12)	0.0610 (5)	
H6A	0.8231	0.4748	0.0887	0.073*	
H6B	0.8393	0.3460	0.1026	0.073*	
C7	0.70189 (12)	0.50185 (14)	0.15283 (10)	0.0462 (4)	
C8	0.74248 (12)	0.60625 (14)	0.16529 (11)	0.0502 (4)	
H8	0.8018	0.6143	0.1659	0.060*	
C9	0.69411 (12)	0.69830 (14)	0.17677 (10)	0.0475 (4)	
H9	0.7217	0.7680	0.1845	0.057*	
C10	0.60613 (11)	0.69016 (13)	0.17709 (9)	0.0416 (4)	
C11	0.56757 (12)	0.58392 (14)	0.16520 (10)	0.0481 (4)	
H11	0.5089	0.5751	0.1662	0.058*	
C12	0.61385 (12)	0.49206 (14)	0.15200 (10)	0.0483 (4)	
H12	0.5856	0.4228	0.1424	0.058*	
C13	0.55467 (11)	0.79052 (13)	0.19559 (9)	0.0425 (4)	
C14	0.59517 (12)	0.90292 (14)	0.18369 (11)	0.0479 (4)	
C15	0.60298 (15)	0.94869 (17)	0.11487 (13)	0.0653 (5)	
H15	0.5828	0.9100	0.0677	0.078*	
C16	0.64175 (17)	1.05427 (19)	0.11778 (17)	0.0792 (7)	
H16	0.6484	1.0859	0.0721	0.095*	
C17	0.67034 (16)	1.11222 (18)	0.18756 (18)	0.0803 (7)	
H17	0.6949	1.1832	0.1881	0.096*	
C18	0.66323 (14)	1.06687 (17)	0.25624 (16)	0.0695 (6)	
H18	0.6832	1.1063	0.3031	0.083*	
C19	0.62587 (11)	0.96158 (15)	0.25474 (11)	0.0510 (4)	
C20	0.60928 (11)	0.89381 (15)	0.31708 (10)	0.0487 (4)	
C21	0.62633 (13)	0.91414 (19)	0.39751 (12)	0.0648 (6)	
H21	0.6534	0.9805	0.4196	0.078*	
C22	0.60246 (15)	0.8344 (2)	0.44319 (12)	0.0739 (7)	
H22	0.6131	0.8474	0.4966	0.089*	
C23	0.56317 (14)	0.7359 (2)	0.41104 (12)	0.0706 (6)	
H23	0.5476	0.6829	0.4430	0.085*	
C24	0.54639 (13)	0.71426 (17)	0.33173 (10)	0.0564 (5)	
H24	0.5206	0.6469	0.3105	0.068*	
C25	0.56844 (11)	0.79406 (15)	0.28465 (9)	0.0450 (4)	
C26	0.45506 (12)	0.79041 (13)	0.14816 (9)	0.0432 (4)	
C27	0.42460 (12)	0.74664 (15)	0.07270 (10)	0.0492 (4)	
H27	0.4653	0.7141	0.0503	0.059*	
C28	0.33544 (13)	0.75042 (16)	0.03025 (10)	0.0525 (4)	
H28	0.3167	0.7195	−0.0198	0.063*	
C29	0.27377 (12)	0.79966 (15)	0.06131 (10)	0.0496 (4)	
C30	0.30209 (13)	0.84633 (17)	0.13524 (11)	0.0575 (5)	
H30	0.2614	0.8809	0.1567	0.069*	
C31	0.39187 (13)	0.84105 (16)	0.17724 (11)	0.0539 (5)	
H31	0.4104	0.8729	0.2270	0.065*	
C32	0.11751 (13)	0.8322 (2)	0.04370 (12)	0.0643 (5)	
H32A	0.1150	0.9132	0.0449	0.077*	
H32B	0.1277	0.8044	0.0968	0.077*	
C33	0.03097 (14)	0.78602 (18)	−0.00918 (12)	0.0592 (5)	
C34	−0.12991 (15)	0.7891 (2)	−0.02384 (14)	0.0750 (6)	
C35	−0.16201 (17)	0.8197 (2)	−0.10832 (14)	0.0878 (7)	
H35A	−0.1587	0.8994	−0.1137	0.132*	
H35B	−0.2228	0.7957	−0.1300	0.132*	
H35C	−0.1253	0.7838	−0.1359	0.132*	
C36	−0.13214 (19)	0.6639 (3)	−0.0107 (2)	0.1187 (11)	
H36A	−0.0988	0.6264	−0.0407	0.178*	
H36B	−0.1930	0.6383	−0.0272	0.178*	
H36C	−0.1061	0.6478	0.0441	0.178*	
C37	−0.18185 (19)	0.8536 (3)	0.02153 (19)	0.1255 (13)	
H37A	−0.1788	0.9322	0.0114	0.188*	
H37B	−0.1565	0.8396	0.0767	0.188*	
H37C	−0.2431	0.8298	0.0052	0.188*	

### Atomic displacement parameters (Å^2^)

**Table:**

	*U*^11^	*U*^22^	*U*^33^	*U*^12^	*U*^13^	*U*^23^
O1	0.0500 (8)	0.0662 (9)	0.0737 (9)	−0.0015 (7)	0.0056 (7)	0.0012 (7)
O2	0.0921 (13)	0.1007 (13)	0.0749 (11)	−0.0092 (10)	0.0100 (9)	0.0199 (9)
O3	0.0519 (8)	0.0475 (7)	0.0819 (10)	−0.0023 (6)	0.0205 (7)	−0.0109 (6)
O4	0.0434 (8)	0.1055 (11)	0.0512 (8)	−0.0002 (7)	0.0057 (6)	−0.0054 (7)
O5	0.0602 (10)	0.0932 (11)	0.0883 (11)	−0.0021 (8)	0.0097 (8)	−0.0280 (9)
O6	0.0460 (8)	0.1080 (12)	0.0651 (9)	−0.0146 (8)	0.0144 (7)	−0.0173 (8)
C1	0.0516 (12)	0.0665 (13)	0.0878 (16)	0.0021 (10)	−0.0014 (11)	−0.0100 (12)
C2	0.0738 (17)	0.0862 (18)	0.117 (2)	0.0229 (14)	−0.0044 (15)	−0.0026 (16)
C3	0.0646 (16)	0.107 (2)	0.139 (3)	−0.0204 (15)	0.0059 (16)	0.0190 (19)
C4	0.098 (2)	0.106 (2)	0.118 (2)	−0.0049 (17)	0.0063 (18)	−0.0490 (18)
C5	0.0571 (13)	0.0506 (11)	0.0729 (14)	0.0062 (10)	0.0137 (11)	−0.0025 (10)
C6	0.0518 (12)	0.0581 (12)	0.0717 (13)	0.0000 (9)	0.0158 (10)	−0.0133 (10)
C7	0.0487 (10)	0.0462 (10)	0.0432 (9)	−0.0006 (8)	0.0127 (8)	−0.0013 (7)
C8	0.0403 (9)	0.0505 (10)	0.0588 (11)	−0.0065 (8)	0.0130 (8)	−0.0034 (8)
C9	0.0445 (10)	0.0428 (9)	0.0535 (10)	−0.0077 (8)	0.0121 (8)	−0.0045 (7)
C10	0.0434 (9)	0.0458 (9)	0.0346 (8)	−0.0039 (7)	0.0101 (7)	−0.0002 (7)
C11	0.0443 (10)	0.0504 (10)	0.0522 (10)	−0.0075 (8)	0.0179 (8)	0.0018 (8)
C12	0.0520 (11)	0.0418 (9)	0.0515 (10)	−0.0110 (8)	0.0158 (8)	−0.0006 (8)
C13	0.0428 (9)	0.0464 (9)	0.0375 (8)	−0.0034 (7)	0.0106 (7)	−0.0009 (7)
C14	0.0430 (10)	0.0452 (9)	0.0550 (10)	0.0005 (8)	0.0136 (8)	0.0014 (8)
C15	0.0708 (14)	0.0592 (12)	0.0691 (13)	−0.0016 (11)	0.0255 (11)	0.0094 (10)
C16	0.0752 (16)	0.0629 (14)	0.107 (2)	0.0021 (12)	0.0389 (15)	0.0285 (14)
C17	0.0579 (14)	0.0453 (11)	0.135 (2)	−0.0059 (10)	0.0247 (15)	0.0053 (14)
C18	0.0500 (12)	0.0490 (11)	0.1032 (18)	−0.0006 (9)	0.0127 (11)	−0.0093 (12)
C19	0.0362 (9)	0.0443 (9)	0.0680 (12)	0.0031 (8)	0.0084 (8)	−0.0087 (8)
C20	0.0336 (9)	0.0602 (11)	0.0476 (10)	0.0085 (8)	0.0047 (7)	−0.0084 (8)
C21	0.0447 (11)	0.0813 (14)	0.0588 (12)	0.0109 (10)	0.0003 (9)	−0.0241 (11)
C22	0.0542 (13)	0.120 (2)	0.0421 (11)	0.0176 (13)	0.0060 (9)	−0.0052 (12)
C23	0.0563 (13)	0.1094 (18)	0.0446 (11)	0.0073 (12)	0.0125 (10)	0.0115 (12)
C24	0.0486 (11)	0.0740 (13)	0.0450 (10)	−0.0004 (9)	0.0113 (8)	0.0061 (9)
C25	0.0362 (9)	0.0572 (10)	0.0394 (9)	0.0049 (8)	0.0079 (7)	−0.0027 (7)
C26	0.0446 (10)	0.0449 (9)	0.0382 (8)	−0.0030 (7)	0.0094 (7)	0.0015 (7)
C27	0.0494 (10)	0.0599 (11)	0.0394 (9)	−0.0023 (9)	0.0150 (8)	−0.0008 (8)
C28	0.0520 (11)	0.0665 (12)	0.0357 (9)	−0.0071 (9)	0.0078 (8)	−0.0031 (8)
C29	0.0437 (10)	0.0577 (11)	0.0436 (9)	−0.0032 (8)	0.0069 (8)	0.0054 (8)
C30	0.0499 (11)	0.0670 (12)	0.0523 (11)	0.0094 (9)	0.0102 (9)	−0.0068 (9)
C31	0.0525 (11)	0.0610 (11)	0.0436 (10)	0.0021 (9)	0.0069 (8)	−0.0100 (8)
C32	0.0482 (11)	0.0828 (14)	0.0594 (12)	−0.0057 (10)	0.0122 (9)	−0.0034 (10)
C33	0.0505 (12)	0.0699 (13)	0.0542 (11)	−0.0048 (10)	0.0108 (9)	0.0029 (10)
C34	0.0485 (12)	0.1056 (19)	0.0681 (14)	−0.0184 (12)	0.0128 (10)	−0.0076 (12)
C35	0.0669 (15)	0.111 (2)	0.0733 (16)	0.0061 (14)	0.0012 (12)	−0.0057 (14)
C36	0.0694 (18)	0.135 (3)	0.134 (3)	−0.0339 (18)	0.0025 (17)	0.039 (2)
C37	0.0640 (17)	0.215 (4)	0.106 (2)	−0.024 (2)	0.0379 (17)	−0.049 (2)

### Geometric parameters (Å, °)

**Table:**

O1—C5	1.331 (2)	C16—C17	1.378 (4)
O1—C1	1.482 (3)	C16—H16	0.9300
O2—C5	1.195 (2)	C17—C18	1.373 (3)
O3—C7	1.376 (2)	C17—H17	0.9300
O3—C6	1.415 (2)	C18—C19	1.384 (3)
O4—C29	1.374 (2)	C18—H18	0.9300
O4—C32	1.407 (2)	C19—C20	1.459 (3)
O5—C33	1.189 (2)	C20—C25	1.395 (2)
O6—C33	1.329 (2)	C20—C21	1.400 (3)
O6—C34	1.486 (3)	C21—C22	1.374 (3)
C1—C2	1.504 (3)	C21—H21	0.9300
C1—C3	1.509 (4)	C22—C23	1.373 (3)
C1—C4	1.514 (3)	C22—H22	0.9300
C2—H2A	0.9600	C23—C24	1.384 (3)
C2—H2B	0.9600	C23—H23	0.9300
C2—H2C	0.9600	C24—C25	1.379 (2)
C3—H3A	0.9600	C24—H24	0.9300
C3—H3B	0.9600	C26—C31	1.384 (2)
C3—H3C	0.9600	C26—C27	1.390 (2)
C4—H4A	0.9600	C27—C28	1.379 (3)
C4—H4B	0.9600	C27—H27	0.9300
C4—H4C	0.9600	C28—C29	1.378 (3)
C5—C6	1.508 (3)	C28—H28	0.9300
C6—H6A	0.9700	C29—C30	1.378 (3)
C6—H6B	0.9700	C30—C31	1.386 (3)
C7—C12	1.379 (3)	C30—H30	0.9300
C7—C8	1.388 (2)	C31—H31	0.9300
C8—C9	1.384 (2)	C32—C33	1.509 (3)
C8—H8	0.9300	C32—H32A	0.9700
C9—C10	1.382 (2)	C32—H32B	0.9700
C9—H9	0.9300	C34—C35	1.486 (3)
C10—C11	1.394 (2)	C34—C37	1.515 (4)
C10—C13	1.533 (2)	C34—C36	1.515 (4)
C11—C12	1.373 (2)	C35—H35A	0.9600
C11—H11	0.9300	C35—H35B	0.9600
C12—H12	0.9300	C35—H35C	0.9600
C13—C14	1.526 (2)	C36—H36A	0.9600
C13—C25	1.538 (2)	C36—H36B	0.9600
C13—C26	1.540 (2)	C36—H36C	0.9600
C14—C15	1.381 (3)	C37—H37A	0.9600
C14—C19	1.403 (2)	C37—H37B	0.9600
C15—C16	1.394 (3)	C37—H37C	0.9600
C15—H15	0.9300		
			
C5—O1—C1	123.30 (17)	C17—C18—H18	120.5
C7—O3—C6	116.90 (14)	C19—C18—H18	120.5
C29—O4—C32	119.62 (15)	C18—C19—C14	120.14 (19)
C33—O6—C34	121.20 (17)	C18—C19—C20	131.20 (19)
O1—C1—C2	108.35 (18)	C14—C19—C20	108.65 (15)
O1—C1—C3	101.74 (19)	C25—C20—C21	119.90 (18)
C2—C1—C3	111.9 (2)	C25—C20—C19	108.74 (15)
O1—C1—C4	110.7 (2)	C21—C20—C19	131.35 (18)
C2—C1—C4	112.6 (2)	C22—C21—C20	118.9 (2)
C3—C1—C4	110.9 (2)	C22—C21—H21	120.5
C1—C2—H2A	109.5	C20—C21—H21	120.5
C1—C2—H2B	109.5	C23—C22—C21	120.89 (19)
H2A—C2—H2B	109.5	C23—C22—H22	119.6
C1—C2—H2C	109.5	C21—C22—H22	119.6
H2A—C2—H2C	109.5	C22—C23—C24	120.9 (2)
H2B—C2—H2C	109.5	C22—C23—H23	119.6
C1—C3—H3A	109.5	C24—C23—H23	119.6
C1—C3—H3B	109.5	C25—C24—C23	119.1 (2)
H3A—C3—H3B	109.5	C25—C24—H24	120.5
C1—C3—H3C	109.5	C23—C24—H24	120.5
H3A—C3—H3C	109.5	C24—C25—C20	120.31 (16)
H3B—C3—H3C	109.5	C24—C25—C13	128.64 (16)
C1—C4—H4A	109.5	C20—C25—C13	111.04 (15)
C1—C4—H4B	109.5	C31—C26—C27	116.57 (16)
H4A—C4—H4B	109.5	C31—C26—C13	120.93 (14)
C1—C4—H4C	109.5	C27—C26—C13	122.37 (15)
H4A—C4—H4C	109.5	C28—C27—C26	121.49 (17)
H4B—C4—H4C	109.5	C28—C27—H27	119.3
O2—C5—O1	126.8 (2)	C26—C27—H27	119.3
O2—C5—C6	124.7 (2)	C29—C28—C27	120.61 (16)
O1—C5—C6	108.45 (18)	C29—C28—H28	119.7
O3—C6—C5	113.08 (17)	C27—C28—H28	119.7
O3—C6—H6A	109.0	O4—C29—C28	115.23 (16)
C5—C6—H6A	109.0	O4—C29—C30	125.40 (17)
O3—C6—H6B	109.0	C28—C29—C30	119.37 (17)
C5—C6—H6B	109.0	C29—C30—C31	119.20 (17)
H6A—C6—H6B	107.8	C29—C30—H30	120.4
O3—C7—C12	115.82 (15)	C31—C30—H30	120.4
O3—C7—C8	125.03 (16)	C26—C31—C30	122.72 (16)
C12—C7—C8	119.15 (16)	C26—C31—H31	118.6
C9—C8—C7	119.55 (17)	C30—C31—H31	118.6
C9—C8—H8	120.2	O4—C32—C33	107.86 (17)
C7—C8—H8	120.2	O4—C32—H32A	110.1
C10—C9—C8	122.25 (16)	C33—C32—H32A	110.1
C10—C9—H9	118.9	O4—C32—H32B	110.1
C8—C9—H9	118.9	C33—C32—H32B	110.1
C9—C10—C11	116.78 (15)	H32A—C32—H32B	108.4
C9—C10—C13	122.20 (14)	O5—C33—O6	126.30 (19)
C11—C10—C13	120.87 (15)	O5—C33—C32	124.5 (2)
C12—C11—C10	121.80 (16)	O6—C33—C32	109.15 (18)
C12—C11—H11	119.1	O6—C34—C35	110.30 (19)
C10—C11—H11	119.1	O6—C34—C37	102.72 (19)
C11—C12—C7	120.43 (16)	C35—C34—C37	109.9 (2)
C11—C12—H12	119.8	O6—C34—C36	108.6 (2)
C7—C12—H12	119.8	C35—C34—C36	112.5 (2)
C14—C13—C10	113.09 (13)	C37—C34—C36	112.3 (3)
C14—C13—C25	100.45 (13)	C34—C35—H35A	109.5
C10—C13—C25	108.69 (13)	C34—C35—H35B	109.5
C14—C13—C26	108.63 (13)	H35A—C35—H35B	109.5
C10—C13—C26	113.10 (13)	C34—C35—H35C	109.5
C25—C13—C26	112.24 (13)	H35A—C35—H35C	109.5
C15—C14—C19	120.51 (17)	H35B—C35—H35C	109.5
C15—C14—C13	128.38 (17)	C34—C36—H36A	109.5
C19—C14—C13	111.11 (15)	C34—C36—H36B	109.5
C14—C15—C16	118.5 (2)	H36A—C36—H36B	109.5
C14—C15—H15	120.8	C34—C36—H36C	109.5
C16—C15—H15	120.8	H36A—C36—H36C	109.5
C17—C16—C15	120.7 (2)	H36B—C36—H36C	109.5
C17—C16—H16	119.7	C34—C37—H37A	109.5
C15—C16—H16	119.7	C34—C37—H37B	109.5
C18—C17—C16	121.1 (2)	H37A—C37—H37B	109.5
C18—C17—H17	119.5	C34—C37—H37C	109.5
C16—C17—H17	119.5	H37A—C37—H37C	109.5
C17—C18—C19	119.1 (2)	H37B—C37—H37C	109.5
			
C5—O1—C1—C2	65.4 (3)	C18—C19—C20—C21	−0.3 (3)
C5—O1—C1—C3	−176.6 (2)	C14—C19—C20—C21	−179.32 (18)
C5—O1—C1—C4	−58.6 (3)	C25—C20—C21—C22	0.2 (3)
C1—O1—C5—O2	8.0 (3)	C19—C20—C21—C22	179.34 (18)
C1—O1—C5—C6	−171.80 (16)	C20—C21—C22—C23	0.5 (3)
C7—O3—C6—C5	76.5 (2)	C21—C22—C23—C24	−0.1 (3)
O2—C5—C6—O3	32.7 (3)	C22—C23—C24—C25	−1.0 (3)
O1—C5—C6—O3	−147.43 (16)	C23—C24—C25—C20	1.7 (3)
C6—O3—C7—C12	168.15 (16)	C23—C24—C25—C13	−178.61 (17)
C6—O3—C7—C8	−11.7 (3)	C21—C20—C25—C24	−1.3 (3)
O3—C7—C8—C9	179.95 (16)	C19—C20—C25—C24	179.36 (16)
C12—C7—C8—C9	0.1 (3)	C21—C20—C25—C13	178.93 (15)
C7—C8—C9—C10	0.7 (3)	C19—C20—C25—C13	−0.38 (19)
C8—C9—C10—C11	0.0 (3)	C14—C13—C25—C24	−179.04 (17)
C8—C9—C10—C13	175.55 (15)	C10—C13—C25—C24	−60.1 (2)
C9—C10—C11—C12	−1.5 (2)	C26—C13—C25—C24	65.7 (2)
C13—C10—C11—C12	−177.08 (15)	C14—C13—C25—C20	0.67 (17)
C10—C11—C12—C7	2.3 (3)	C10—C13—C25—C20	119.58 (15)
O3—C7—C12—C11	178.61 (15)	C26—C13—C25—C20	−114.56 (15)
C8—C7—C12—C11	−1.5 (3)	C14—C13—C26—C31	−81.16 (19)
C9—C10—C13—C14	21.0 (2)	C10—C13—C26—C31	152.41 (16)
C11—C10—C13—C14	−163.57 (15)	C25—C13—C26—C31	29.0 (2)
C9—C10—C13—C25	−89.59 (18)	C14—C13—C26—C27	94.57 (18)
C11—C10—C13—C25	85.79 (18)	C10—C13—C26—C27	−31.9 (2)
C9—C10—C13—C26	145.05 (16)	C25—C13—C26—C27	−155.27 (16)
C11—C10—C13—C26	−39.6 (2)	C31—C26—C27—C28	−2.1 (3)
C10—C13—C14—C15	64.2 (2)	C13—C26—C27—C28	−177.98 (16)
C25—C13—C14—C15	179.82 (19)	C26—C27—C28—C29	1.0 (3)
C26—C13—C14—C15	−62.3 (2)	C32—O4—C29—C28	171.85 (18)
C10—C13—C14—C19	−116.39 (16)	C32—O4—C29—C30	−8.1 (3)
C25—C13—C14—C19	−0.75 (18)	C27—C28—C29—O4	−179.37 (16)
C26—C13—C14—C19	117.17 (15)	C27—C28—C29—C30	0.6 (3)
C19—C14—C15—C16	−0.2 (3)	O4—C29—C30—C31	178.94 (18)
C13—C14—C15—C16	179.23 (18)	C28—C29—C30—C31	−1.1 (3)
C14—C15—C16—C17	−1.0 (3)	C27—C26—C31—C30	1.6 (3)
C15—C16—C17—C18	1.4 (4)	C13—C26—C31—C30	177.61 (17)
C16—C17—C18—C19	−0.6 (3)	C29—C30—C31—C26	−0.1 (3)
C17—C18—C19—C14	−0.5 (3)	C29—O4—C32—C33	−161.50 (17)
C17—C18—C19—C20	−179.45 (19)	C34—O6—C33—O5	7.3 (3)
C15—C14—C19—C18	0.9 (3)	C34—O6—C33—C32	−172.50 (19)
C13—C14—C19—C18	−178.58 (16)	O4—C32—C33—O5	8.2 (3)
C15—C14—C19—C20	−179.94 (17)	O4—C32—C33—O6	−171.93 (16)
C13—C14—C19—C20	0.6 (2)	C33—O6—C34—C35	−67.6 (3)
C18—C19—C20—C25	178.91 (19)	C33—O6—C34—C37	175.2 (2)
C14—C19—C20—C25	−0.12 (19)	C33—O6—C34—C36	56.1 (3)
